# Platelet-rich plasma improves pain and function in knee osteoarthritis: a retrospective study

**DOI:** 10.3389/fphys.2025.1678037

**Published:** 2025-10-29

**Authors:** Yi-Ting Sun, Xiao-Na Xiang, Jie Yang, Jia-Lei Peng, Hong-Chen He

**Affiliations:** ^1^ Rehabilitation Medicine Center and Institute of Rehabilitation Medicine, West China Hospital, Sichuan University, Chengdu, China; ^2^ School of Rehabilitation Sciences, West China School of Medicine, Sichuan University, Chengdu, China; ^3^ Key Laboratory of Rehabilitation Medicine in Sichuan Province, West China Hospital, Sichuan University, Chengdu, China; ^4^ Department of Rehabilitation Medicine, West China Hospital, Sichuan University/West China School of Nursing, Sichuan University, Chengdu, China

**Keywords:** platelet-rich plasma, knee osteoarthritis, visual analog scale, WOMAC, treatment outcomes, predictors

## Abstract

**Introduction:**

This study aimed to evaluate the efficacy of platelet-rich plasma (PRP) in treating knee osteoarthritis (KOA) and the effects of baseline characteristics and PRP intervention parameters on treatment outcomes.

**Methods:**

Overall, 140 individuals diagnosed with KOA who received PRP injections and completed a 6-month follow-up period were enrolled in this retrospective analysis. Knee pain and functional outcomes were assessed using the Visual Analog Scale (VAS) and the Western Ontario and McMaster Universities Osteoarthritis Index (WOMAC). Based on the Minimal Clinically Important Difference (MCID) in outcomes, the participants were divided into effective and ineffective groups. Using multivariable logistic regression to explore factors influencing treatment outcomes, we compared the effective and ineffective groups to identify predictors of response to PRP therapy.

**Results:**

At 6 months, the median (IQR) VAS score significantly decreased from 66.5 (27) to 24 (34) (95% CI = −38 to −30.5), p < 0.001), and WOMAC scores improved from 29 (22) to 12 (14) (95% CI = −16.5 to −12), p < 0.001). Five mild adverse events were reported. Multivariate analysis indicated that only the number of injections significantly influenced VAS outcomes (OR = 4.285, 95% CI: 1.586–11.578, p = 0.004). Regarding WOMAC, multivariate analysis revealed that body mass index (BMI) (OR = 0.867, 95% CI: 0.755–0.995, p = 0.042) and disease duration (OR = 0.905, 95% CI: 0.784–0.989, p = 0.045) significantly affected outcomes. Age, sex, Kellgren-Lawrence (KL) grade, number of PRP injections, and injection frequency did not significantly impact WOMAC scores.

**Conclusion:**

PRP therapy is a safe and effective treatment option for KOA. In this 6-month follow-up investigation, we observed that the number of injections administered affected pain levels, while disease duration and BMI affected knee joint function. Insights from this study may facilitate patient selection and PRP treatment protocol optimization in clinical practice.

## 1 Introduction

Knee osteoarthritis (KOA) is a common musculoskeletal disease that affects approximately 364.58 million people globally and has become a major disabling condition. Notably, female individuals account for approximately two-thirds of the 225 million patients worldwide (Li et al., 2024). Therefore, effective and safe medical treatments for KOA are urgently required. Currently, no disease-modifying drugs have been approved, and existing non-operative therapies have demonstrated only limited benefits and may be associated with serious adverse effects (Arden et al., 2020; Kolasinski et al., 2020; McAlindon et al., 2014).

Platelet-rich plasma (PRP), a safe autologous blood product rich in various growth factors and cytokines, may influence the biological mechanisms underlying KOA progression and symptom manifestation (Fice et al., 2019; Gupta et al., 2020; Liu et al., 2023). Recently, PRP has gained popularity as a treatment option for individuals with KOA (Fice et al., 2019; Magruder et al., 2023; Werner et al., 2020), especially for pain management. For intra-articular injections, a meta-analysis indicated that for individuals with KOA, the combination therapy of PRP and hyaluronic acid is safe and also superior to PRP monotherapy in terms of pain relief and functional enhancement (Du and Liang, 2025). Additionally, several systematic reviews reported that PRP was associated with more favorable pain and function outcomes compared to intra-articular corticosteroids (Wang et al., 2024) or intra-articular hyaluronic acid (Hohmann et al., 2020), particularly in the long-term management of KOA (Patel et al., 2013; Bensa et al., 2025). However, a systematic review (Costa et al., 2022) indicated that there was limited evidence to support its clinical benefits. According to KOA clinical guidelines (Kolasinski et al., 2020; McAlindon et al., 2014), PRP is not recommended due to very low-certainty evidence and heterogeneity. Therefore, rigorous studies to demonstrate its effectiveness are required. Previous studies (Karaborklu et al., 2023; Saraf et al., 2023; Tao et al., 2023; Xiong et al., 2017) reported how individual characteristics such as age, sex, and PRP dosage affected the clinical outcomes of PRP treatment. These factors may potentially account for the heterogeneity observed in PRP treatment efficacy. Therefore, a large-scale retrospective study is needed to clarify these factors and their impact on treatment outcomes.

Within a 6-month follow-up period, this study aimed to evaluate the clinical effectiveness of PRP injections in relieving pain and enhancing joint function for individuals with KOA. Furthermore, we aimed to investigate whether individual characteristics (e.g., age, sex, disease duration, body mass index (BMI), and Kellgren–Lawrence (KL) grade) and PRP intervention parameters (e.g., number and frequency of injections) could influence the outcomes. The findings of this study will bridge this gap and provide more personalized recommendations for individuals with KOA of varying severities.

## 2 Methods

### 2.1 Study design and setting

This retrospective study was registered with the Chinese Clinical Trial Registry (Registration Number: ChiCTR2500103500). The data collection process adhered strictly to the ethical guidelines outlined by the World Medical Association’s Declaration of Helsinki. The study followed the STROBE guidelines (Supplementary Table S1) and was approved by the Biomedical Research Ethics Committee of West China Hospital, Sichuan University (Approval No. 691, reviewed in 2024). Given the retrospective nature of the study, the committee granted an exemption from obtaining informed consent. All data were kept strictly confidential and used exclusively for the purposes of this study.

### 2.2 Participants

Eligible participants were individuals aged 18–80 years who were diagnosed with KOA and received PRP therapy between January 2022 and November 2023 at a rehabilitation medical center. The inclusion criteria followed the national clinical guidelines (Zhu et al., 2023) and required recurrent knee pain in the previous month along with at least two of the following: (1) Radiographic findings (from standing or weight-bearing views) indicating joint space narrowing, subchondral sclerosis and/or cystic changes, and osteophyte formation at the joint margins; (2) age ≥50 years; (3) morning stiffness lasting ≤30 min; (4) audible joint crepitus during movement. Additional inclusion criteria included a VAS score of ≥40/100 and receiving at least one PRP injection. The exclusion criteria included individuals diagnosed with other lower limb disorders that affected daily activities or osteoarthritis of other joints (such as the hip or ankle), PRP injection duration of less than 8 weeks (Gobbi et al., 2015), or those receiving other biological treatments (such as stem cell therapy or systemic immunosuppressive medications) within the past year.

### 2.3 PRP preparation and injection

#### 2.3.1 PRP preparation

PRP was prepared following a previously published protocol (Liu et al., 2023). A total of 46 mL blood was drawn from the median cubital vein, ensuring that 45 mL of them for preparing PRP for one knee, and 1 mL of blood was used for laboratory testing. The EasyPRP Centrifuge, model PRP520R (EasyPRP, China), was used to prepare PRP using a two-time centrifugation method. 45 mL of blood was anticoagulated with 5 mL of an anticoagulant agent and then centrifuged at low speed. After removing the supernatant, the sediment was centrifuged again. The final product was approximately 5 mL of leukocyte-poor PRP, and 1 mL of them was used for laboratory testing for comparing the number of platelet concentration to the baseline.

#### 2.3.2 PRP injection

All injection procedures were performed by clinicians with over 5 years of experience in injection therapy. Standardization was ensured through uniform training in PRP preparation and application. All injections were administered under ultrasound guidance (LOGIQ e, GE Healthcare, Milwaukee, Wisconsin, United States), using a probe with frequencies between 7.5 and 14 MHz, to enhance the precision of all injections, minimize variability, and maximize the accuracy of the procedure. Trained operators administered 5 mL of PRP into the affected knee under ultrasound guidance within 30 min of preparation. Blood samples (1 mL of whole blood and 1 mL of PRP) were analyzed. In cases of joint effusion, aspiration via the suprapatellar pouch was performed before PRP injection. Participants were advised to avoid medications for 6 months post-treatment to ensure accurate assessment of PRP efficacy.

### 2.4 Outcomes

The primary outcomes were pain and functional assessments for 6 months after the first injection. Pain was assessed using the VAS, and function was evaluated using the Western Ontario and McMaster Universities Osteoarthritis Index (WOMAC), which is used for overall knee function assessment. The Minimal Clinically Important Difference (MCID) for both VAS (Lee et al., 2003) and WOMAC (Hmamouchi et al., 2012) is approximately 20%. The participants were classified into the effective and the ineffective groups based on the MCID of their VAS or WOMAC. Those with changes beyond the MCID were included in the effective group. The secondary outcome was safety, which was primarily evaluated through adverse event reports during the 6-month follow-up period.

### 2.5 Clinical data

Demographic data of the participants, laboratory results of PRP, and outcomes were obtained from the electronic medical system record (HIS) at a rehabilitation medical center. Data were extracted from the system at baseline and assessed manually at the 6-month follow-up. Specifically, we analyzed whether there were significant differences between the groups in terms of age, disease duration, sex, BMI, KL grade, injection frequency, and number of injections.

### 2.6 Statistical analysis

Statistical analysis was performed using SPSS Statistics, version 27.0 (IBM Corp., Armonk, NY, United States) and R version 4.1.0 (R Core Team, Vienna, Austria). A P-value <0.05 was considered statistically significant. Given the observational nature of our study, we followed the recommendation from a previous study (Ahmad and Halim, 2017) to set the sample size at 5–10 times the number of factors. For normally distributed continuous variables, estimated means and standard deviations were presented. Medians and interquartile ranges (IQR) were used for non-normally distributed variables. Normality was assessed through visual inspection of histograms or normal Q - Q plots and verified using the Kolmogorov–Smirnov test. Baseline between-group differences were determined using the Student’s t-test or Mann–Whitney U test, as appropriate. Categorical variables were analyzed via the chi-square or Fisher’s exact tests. Primary analyses were conducted on an available-case basis, including participants with both baseline and 6-month data; missing outcomes were not imputed. Additionally, a multivariate analysis was conducted with treatment effectiveness (categorized as effective or ineffective) as the dependent variable. Given that the dependent variable is categorical, logistic regression analysis was employed to assess the relationship between the predictors and the outcome of treatment effectiveness. Both continuous (age, BMI, disease duration) and categorical (sex, KL grade, number of injections) predictors were included in the model.

## 3 Results

### 3.1 Participants’ characteristics

**FIGURE 1:**
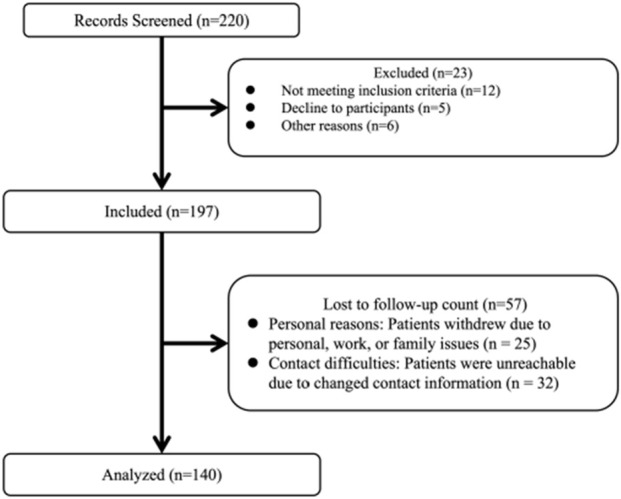
Flow chart.

A total of 220 participants with KOA who received PRP injections participated in this trial. Overall, 23 participants were excluded at screening and 57 were lost to follow-up after 6 months. Finally, 140 individuals were included in this study (Figure 1). The baseline characteristics of those that completed the trial (completers) and those who did not (non-completers) are shown in Table 1. Among the participants, 102 (72.9%) were female and 38 (27.1%) were male. The mean age of the participants was 60.4 ± 10.7 years. The median disease duration was 3 years (IQR: 1.5–6.0 years). Based on the KL grade system, 52 participants (37.1%) were graded as level I, 42 individuals (30%) as level II, 44 individuals (34.1%) as level III, and 2 individuals (1.4%) as level IV. As shown in Table 2, the platelet concentration in the prepared PRP was approximately 2.91 times that of whole blood.

**TABLE 1:** Baseline characteristics of completers and non-completers.

Variable	Complete (*N* = 140)	Non-complete (*N* = 57)	Statistic value	*P* value
Age (years)	61 (16)	60 (15)	Z = −0.932	0.351
M (IQR)				
Disease duration (years)	3 (4.5)	2 (4.13)	Z = −1.171	0.241
M (IQR)				
Sex				
Male	38 (27.14%)	13 (22.81%)	χ^2^ = 0.397	0.529
Female	102 (72.85%)	44 (77.19%)		
BMI	23.48 (4.04)	24.69 (4.4)	Z = −2.458	0.014*
K-L grade				
I	52 (37.14%)	22 (38.61%)	χ^2^ = 0.096	0.992
II	42 (30%)	17 (29.82%)		
III	44 (31.43%)	17 (29.82%)		
IV	2 (1.43%)	1 (1.75%)		
Injection frequency				
1 injection/month	93 (%)	30 (%)	χ^2^ = 3.288	0.07
>1 injection/month	47 (%)	27 (%)		
Number of injections				
1	37 (%)	14 (%)	χ^2^ = 0.074	0.786
>1	103 (%)	33 (%)		
VAS	66.5 (27)	66 (18)	Z = −0.834	0.404
WOMAC	29 (22)	28 (11)	Z = −0.181	0.857

M, median; IQR, interquartile range; BMI, body mass index; K–L grade, Kellgren–Lawrence grade; VAS, visual analog scale; WOMAC, the Western Ontario and McMaster Universities Osteoarthritis Index; *P < 0.05.

**TABLE 2:** PRP preparation data (M, (IQR)).

Blood components	Whole blood (× 10^9^/L)	PRP (× 10^9^/L)
Platelet	194 (158, 228)	565 (300, 828)
Red blood cells	4.48 (4.23, 4.71)	1.87 (0.42, 4.02)
White blood cells	5.35 (4.56, 6.22)	6.53 (4.39, 16.28)

PRP, platelet-rich plasma; M, median; IQR, interquartile range.

### 3.2 Within-group analysis

The median (IQR) VAS score decreased from 66.5 (Buendía-López et al., 2018) to 24 (Dhillon et al., 2017) at 6 months (Z = −10.159, r = −0.86, 95% CI = −38 to −30.5), p < 0.001). In terms of the WOMAC score, a significant improvement was observed as the score declined from 29 (Gobbi et al., 2015) to 12 (Bensa et al., 2025) after 6 months (Z = −9.790, r = −0.83, 95% CI = −16.5 to −12), p < 0.001).

### 3.3 PRP treatment effectiveness analysis

To investigate how baseline characteristics and PRP intervention parameters influenced treatment outcomes, the participants were divided into the effective and the ineffective groups based on the MCID criteria for VAS and WOMAC. Among the 140 individuals who received PRP treatment, 114 were classified as responders based on the VAS results, while 26 were classified as non-responders; regarding WOMAC, 109 were responders and 31 were non-responders.

#### 3.3.1 Univariate analysis of PRP treatment effectiveness according to VAS

As shown in Table 3, significant differences were found between the effective and ineffective groups in terms of disease duration, sex distribution, KL grade, and number of injections. Participants in the effective group had a shorter disease duration (2.75 years, IQR: 0.98–5.50) compared to participants in the ineffective group (4.5 years, IQR: 2.5–10.5; Z = −2.088, *P* = 0.037). Regarding sex, within the effective group, 31.6% of the participants were male and 68.4% were female, while in the ineffective group, the corresponding proportions were 7.7% male and 92.3% female (χ^2^ = 6.11, *P* = 0.01). Specifically, 94.7% of male participants demonstrated a treatment response, compared to 76.5% of female participants. In terms of KL grade, a significant difference was found between the effective and ineffective groups (χ^2^ = 0.82, *P* = 0.04). In the effective group, 38.6% of the participants were classified as grade I, 31.6% as grade II, and 29.8% as grade III. In contrast, in the ineffective group, 30.8% were grade I, 23.1% were grade II, 38.5% were grade III, and 7.69% were grade IV. Additionally, the number of injections also appeared to exert a significant influence (χ^2^ = 5.2, *P* = 0.02). In the effective group, 21.9% of participants received a single injection, while 78.1% received repeated injections. Conversely, in the ineffective group, 46.2% received one injection and 53.8% received more than one injection. However, no significant differences were observed between the two groups in terms of age and injection frequency.

**TABLE 3:** Univariate analysis of PRP for KOA after 6 months of follow-up according to VAS.

Variable	Efficient (*N* = 114)	Nonefficient (*N* = 26)	Statistic value	*P* value
Age (years)	60 (51.75, 69)	63.5 (59.5, 69.25)	Z = −1.939	0.053
M (IQR)				
Disease duration (years)	2.75 (0.98, 5.50)	4.5 (2.5, 10.5)	Z = −2.088	0.037*
M (IQR)				
Sex				
Male	36 (31.6%)	2 (7.69%)	χ^2^ = 6.11	0.01*
Female	78 (68.4%)	24 (92.31%)		
BMI	23.44 (3.74)	23.71 (4.84)	Z = −0.059	0.953
K–L grade				
I	44 (38.6%)	8 (30.77%)	χ^2^ = 8.02	0.04*
II	36 (31.6%)	6 (23.08%)		
III	34 (29.8%)	10 (38.46%)		
IV	0 (0%)	2 (7.69%)		
Injection frequency				
1 injection/month	71 (62.3%)	22 (84.62%)	χ^2^ = 3.79	0.052
>1 injection/month	43 (37.7%)	4 (15.38%)		
Number of injections				
1	25 (21.9%)	12 (46.2%)	χ^2^ = 5.20	0.02*
>1	89 (78.1%)	14 (53.8%)		

M, median; IQR, interquartile range; VAS, visual analog scale; K–L grade, Kellgren–Lawrence grade; *P < 0.05, **P < 0.01.

#### 3.3.2 Multivariate analysis of PRP treatment effectiveness according to VAS

**FIGURE 2:**
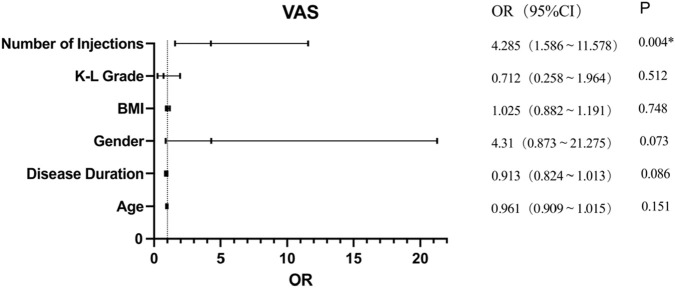
Multivariate analysis of PRP treatment effectiveness according to VAS.

Univariate analysis showed that disease duration, sex, K-L grade, and number of injections influenced PRP treatment effectiveness according to VAS; however, further analysis of its effect using logistic regression analysis showed that only the number of injections (OR = 4.285, 95% CI: 1.586–11.578, p = 0.004) significantly affected PRP treatment effectiveness assessed using VAS scores (Figure 2). This suggests that repeated PRP injections are approximately four times more effective than a single injection in terms of VAS scores.

#### 3.3.3 Univariate analysis of PRP treatment effectiveness according to WOMAC

As shown in Table 4, univariate analysis of WOMAC scores also indicated a significant difference in disease duration between the effective and ineffective groups. The effective group had a shorter disease duration (2.5 years, IQR: 0.87–5.5) compared to the ineffective group (3 years, IQR: 2.5–7.5; Z = −1.993, *P* = 0.046). No significant differences were found between the effective and ineffective groups in terms of age, sex, KL grade, injection frequency, and the number of injections.

**TABLE 4:** Univariate analysis of PRP for KOA after 6 months of follow-up according to WOMAC.

Variable	Efficient (*N* = 109)	Nonefficient (*N* = 31)	Statistic value	*P* value
Age (years)	60.20 ± 10.30	61.06 ± 12.08	Z = −0.711	0.477
M^a^ ± SD				
Disease duration (years)	2.5 (0.87, 5.5)	3 (2.5, 7.5)	Z = −1.993	0.046*
M (IQR)				
Sex				
Male	31 (28.4%)	7 (22.6%)	χ^2^ = 0.419	0.517
Female	78 (71.6%)	24 (77.4%)		
BMI	22.1 (3.74)	24.59 (5.68)	Z = −1.566	0.117
K–L grade				
I	41 (37.6%)	11 (35.5%)	χ^2^ = 2.066	0.559
II	35 (32.1%)	7 (22.6%)		
III	33 (30.3%)	11 (35.5%)		
IV	0 (0%)	2 (6.5%)		
Injection frequency				
1 injection/month	70 (64.2%)	23 (74.2%)	χ^2^ = 1.007	0.299
>1 injection/month	39 (35.8%)	8 (25.8%)		
Number of injections				
1	26 (23.9%)	11 (35.5%)	χ^2^ = 1.679	0.195
>1	83 (76.1%)	20 (64.5%)		

a, Mean; b, Median; IQR, interquartile range; WOMAC, the Western Ontario and McMaster Universities Osteoarthritis Index; K–L grade, Kellgren–Lawrence grade; *P < 0.05.

#### 3.3.4 Multivariate analysis of PRP treatment effectiveness according to WOMAC

**FIGURE 3:**
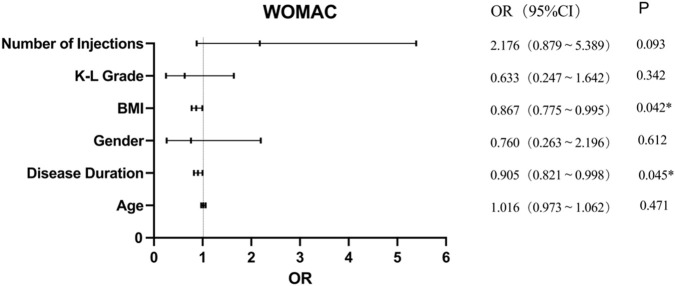
Multivariate analysis of PRP treatment effectiveness according to WOMAC.

Univariate analysis showed that only the disease duration influenced PRP treatment effectiveness according to WOAMC; however, further analysis of its effect using logistic regression analysis showed that BMI (OR = 0.867, 95% CI = 0.755–0.995, p = 0.042) and disease duration (OR = 0.905, 95% CI = 0.784–0.989, p = 0.045) significantly affected PRP treatment effectiveness on WOMAC scores (Figure 3). This revealed that for every 1 kg/m^2^ increase in BMI, the effectiveness of PRP treatment decreased by 13.3%. Similarly, in terms of disease duration, each 1-year increase was associated with a 9.5% reduction in the effectiveness of PRP injections on the WOMAC score. However, age, sex, K-L grade, number of injections of PRP, and injection frequency showed no significant difference.

### 3.4 Safety

By the 6-month follow-up, four participants experienced a total of five adverse events associated with PRP intra-articular injections. The post-injection pain was effectively managed with lidocaine hydrochloride, sodium chloride solution, and diazepam, with the treatment durations ranging from immediate intervention to up to 4 weeks, depending on the characteristics and duration of the adverse reactions. The management of these adverse events is illustrated in Table 5.

**TABLE 5:** Management of adverse events.

No.	Sex	Symptom description	Treatment
1	Male	Persistent pain and joint effusion after the third injection; no treatment by the doctor, resolved within 1 week	After medical examination, no treatment was administered, and the condition resolved within 1 week
2	Female	Pain relief after the 2nd and 3rd injections, followed by knee joint swelling	Injected lidocaine and sodium bicarbonate into the joint cavity, resolved in 3–4 weeks
3	Female	Pain relief after the 2nd and 3rd injections, followed by knee joint swelling	Injected lidocaine and sodium bicarbonate into the joint cavity, resolved in 3–4 weeks
4	Female	Severe pain within 10 h after the first injection	Administered dexamethasone 5 mg on the 4th day after injection, resolved
5	Female	Pain and insomnia due to pain on the night of the injection	Actively treated with cold compresses, pain relief

### 3.5 Unchanged data before and after 6-month PRP injection

Among the 140 participants, four participants had no change in VAS after the intervention, and their information was listed in Table 6. Besides this, six participants had no change in WOMAC after PRP injection, and their information was listed in Table 7.

**TABLE 6:** Individuals with No change in pain.

Case number	Disease duration	Number of injection	Sex	Age (years)	K–L grade	Injection frequency	BMI (kg/m^2^)	Pre VAS	Post VAS	Pre WOMAC	Post WOMAC
01	2.5	3	Female	70	2	3	27.06	72	74	48	45
02	2.5	3	Female	70	3	3	27.06	78	78	50	47
03	10.5	1	Female	61	1	3	18.26	4	6	19	3
04	6.5	2	Female	69	2	3	26.67	65	77	35	33

M, median; IQR, interquartile range; BMI: body mass index, K–L grade, Kellgren–Lawrence grade; VAS, visual analog scale; WOMAC, the Western Ontario and McMaster Universities Osteoarthritis Index; *P < 0.05.

**TABLE 7:** Individuals with No change in WOMAC.

Case number	Disease duration	Number of injection	Sex	Age (Years)	K–L grade	Injection frequency	BMI	Pre VAS	Post VAS	Pre WOMAC	Post WOMAC
05	2.5	1	Female	56	1	3	25.59	89	71	0	37
06	2.5	3	Male	32	1	2	29.39	72	70	55	55
07	3	5	Male	72	2	3	24.39	62	23	25	30
08	0.2	1	Female	52	2	3	18.94	51	25	12	13
09	0.2	1	Female	52	3	3	18.94	72	71	45	47
10	2.5	2	Female	63	1	3	23.71	68	66	43	43

M, median; IQR, interquartile range; BMI: body mass index, K–L grade, Kellgren–Lawrence grade; VAS, visual analog scale; WOMAC, the Western Ontario and McMaster Universities Osteoarthritis Index; *P < 0.05.

## 4 Discussion

This study evaluated the clinical effectiveness of PRP knee injections for KOA, observing significant pain reduction and joint function improvement within 6 months of follow-up, which is consistent with the findings of previous studies (Anil et al., 2021; Buendía-López et al., 2018; Elik et al., 2020; Riboh et al., 2015). Using the MCID, participants were divided into the “effective” and “noneffective” groups based on changes in their VAS and WOMAC scores. Both univariate and multivariate analyses were employed to investigate the factors that influence the effectiveness of PRP injection treatment. The results of our univariate analysis revealed that disease duration, sex, KL grade, and the number of injections administered significantly influenced the effectiveness of PRP treatment. However, multivariate analyses indicated that only the number of injections significantly affected pain levels, while disease duration and BMI influenced WOMAC outcomes. Specifically, PRP appeared to be more effective in pain management in individuals with shorter disease duration, lower BMI, and those who received repeated injections. This discrepancy between the univariate and multivariate analyses may be attributed to the interactions between variables and differences in model assumptions.

Khalilizad et al. (2025) and Vilchez-Cavazos et al. (2019) reported that repeated injections improved joint functionality more than a single injection at 6 months. Our findings are consistent with their observations. Our multivariate analysis, which accounted for potential confounding factors, showed that repeated PRP injections were about four times more effective than a single injection in reducing pain. This relief in pain may be due to the sustained release of growth factors from repeated injections, which promotes cartilage repair and reduces inflammation, thereby enhancing treatment efficacy. Moreover, BMI and disease duration significantly impacted WOMAC scores. These findings are consistent with those of previous studies (Dong et al., 2023; Bansal et al., 2021; Dhillon et al., 2017). In our study, for every 1 kg/m^2^ increase in BMI, the effectiveness of PRP decreased by 13.3%. Similarly, in terms of disease duration, each 1-year increase in disease duration was associated with a 9.5% reduction in the effectiveness of PRP injections on the WOMAC score. The influence of BMI on treatment outcomes may be related to the additional mechanical stress on the knee joint, which could exacerbate cartilage damage and diminish the effectiveness of PRP injections (Yu et al., 2025). Similarly, longer disease duration was associated with more severe cartilage degeneration, which may reduce the potential for repair and thus the effectiveness of PRP treatment (Kon et al., 2009; Kon et al., 2011).

However, in our multivariate analysis, age, sex, KL grade, and injection frequency did not significantly impact the efficacy of PRP treatment. Comparing our results with those previous studies revealed both consistencies and inconsistencies. Regarding age, it is widely recognized that cartilage repair capacity declines with age. However, the effectiveness of PRP may be more closely related to the local concentration of growth factors and the regulation of inflammation rather than to the patient’s age. This aligns with the findings of previous studies that suggest that PRP’s therapeutic potential can be maintained across different age groups due to its ability to enhance local healing factors (Chowdhary et al., 2022; Johnson et al., 2022). Our findings from our correlation analysis indicated an interaction effect with the number of injections. This suggests that the optimal therapeutic outcome may depend on a specific combination of these factors, which is consistent with the conclusions of other studies, highlighting the importance of tailored injection protocols (Tao et al., 2023; Khalilizad et al., 2025). Additionally, regarding KL grade, our analysis did not identify a significant impact on PRP efficacy. One possible reason is that KL grade reflects the structural severity of KOA; however, individuals with the same structural severity can exhibit varying levels of knee function due to individual differences (Sonobe et al., 2024). Therefore, the clinical response to PRP may not be directly correlated with radiographic findings. In contrast, our results for sex differed from those of previous studies. According to a previous research, sex may influence the efficacy of PRP; however, some studies indicate that the response of female subjects to PRP treatment when differ from that of male subjects (Li et al., 2025). Our analysis did not find a significant impact of sex on PRP efficacy. This discrepancy could be attributed to differences in study populations, sample sizes, or the specific methods used to assess PRP effectiveness. Further investigation is warranted to elucidate the role of sex in PRP treatment outcomes.

In terms of cost-effectiveness, PRP injections are relatively inexpensive (Alcerro and Lavernia, 2019; Carducci et al., 2019) compared to joint arthroplasty (Jones et al., 2018) or stem cell therapy (Bendich et al., 2020). We reviewed previous studies that reported on the costs of single and multiple injections, as well as the costs of PRP and hyaluronic acid. Wang et al. (2022) and Karabas and Tezcan (2025) found that single injections of PRP and hyaluronic acid had similar efficacy in improving outcomes. Conversely, Park et al. (2021) demonstrated that single-dose PRP injections were more effective in treating early-stage knee osteoarthritis than hyaluronic acid. Furthermore, Görmeli et al. (2015) reported that repeated PRP injections appeared to be more effective in the long-term management of pain and function compared to single injections of PRP or hyaluronic acid. Despite these findings, few studies have examined the cost-effectiveness of repeated PRP injections, particularly the improvement in pain and function relative to the financial investment when increasing from two to three injections. Further research is needed to determine the value of repeated PRP injection, weighing its added benefits against the increased cost.

### 4.1 Limitations

This study has several limitations. First, the absence of a control group limits our ability to compare the efficacy of PRP injections with other treatment methods, potentially introducing bias. Second, individuals’ compliance with the recommendation to avoid medications for up to 6 months could not be precisely controlled, which may have introduced co-intervention bias. Third, as a single-center trial, our study may be subject to center bias. Multicenter randomized controlled trials are needed to minimize selection bias and confounding factors, as well as to enhance the causal inference of the results.

## 5 Conclusion

This retrospective study analyzed the effectiveness of PRP treatment in individuals with KOA. Our findings showed that the effectiveness of PRP injections for treating KOA was influenced by multiple factors, including the number of injections, BMI, and disease duration. These findings highlight the importance of considering these variables when designing treatment protocols and suggested that personalized treatment strategies may be necessary to optimize outcomes. Future research should further explore the underlying mechanisms and interactions between these factors to refine PRP treatment protocols and improve clinical efficacy.

## Data Availability

The data analyzed in this study is subject to the following licenses/restrictions: In this study, strict restrictions have been implemented on the dataset to ensure the privacy and data security of the participants. All personally identifiable information has been anonymized, and access to the dataset is limited to authorized researchers who are bound by confidentiality agreements. The use of the dataset is strictly confined to the purposes of this study and has been approved by the relevant ethics review committee. To prevent data breaches, we have implemented advanced data security measures. Furthermore, sharing the dataset with third parties is strictly prohibited without the written consent of the data custodian and the ethics review committee. These measures are designed to safeguard the integrity of the research and respect the rights and privacy of the participants. Requests to access these datasets should be directed to hxkfhhc@126.com.

## Abbreviations

KOA, Knee osteoarthritis; PRP, Platelet-rich plasma; OA, Osteoarthritis; MCID, Minimal Clinically Important Difference; VAS, the Visual Analog Scale; WOMAC, the Western Ontario and McMaster Universities Osteoarthritis Index; BMI, Body Mass Index; KL, Kellgren-Lawrence; HIS, the electronic medical system record; IQR, Medians and interquartile ranges.
