# Supplementary material for: Platelet-rich plasma improves pain and function in knee osteoarthritis: a retrospective study
Source: Front Physiol. 2025 Oct 29;16:1678037. doi: 10.3389/fphys.2025.1678037 (PMC12605472; doi:10.3389/fphys.2025.1678037)
Supplement: Supplementary file 1 [file Table1.docx]

STROBE Statement—checklist of items that should be included in reports of observational studies

|  | Item No. | Recommendation | Relevant text from manuscript | |  |
| --- | --- | --- | --- | --- | --- |
| **Title and abstract** | 1 | (*a*) Indicate the study’s design with a commonly used term in the title or the abstract | Platelet-Rich Plasma Improves Pain and Function in Knee Osteoarthritis: A Retrospective Study | |  |
|  |  | (*b*) Provide in the abstract an informative and balanced summary of what was done and what was found | This study aimed to evaluate the efficacy of platelet-rich plasma (PRP) in treating knee osteoarthritis (KOA) and the effects of baseline characteristics and PRP intervention parameters on treatment outcomes. | |  |
| Introduction | | | |  | |
| Background/rationale | 2 | Explain the scientific background and rationale for the investigation being reported | Knee osteoarthritis (KOA) is a common musculoskeletal disease that affects approximately 364.58 million people globally and has become a major disabling condition…  Platelet-rich plasma (PRP), a safe autologous blood product rich in various growth factors and cytokines, may influence the biological mechanisms underlying KOA progression and symptom manifestation (5–7)… | |  |
| Objectives | 3 | State specific objectives, including any prespecified hypotheses | Within a 6-month follow-up period, this study aimed to evaluate the clinical effectiveness of PRP injections in relieving pain and enhancing joint function for individuals with KOA. Furthermore, we aimed to investigate whether individual characteristics (e.g., age, sex, disease duration, body mass index (BMI), and Kellgren–Lawrence (KL) grade) and PRP intervention parameters (e.g., number and frequency of injections) could influence the outcomes. The findings of this study will bridge this gap and provide more personalized recommendations for individuals with KOA of varying severities. | |  |
| Methods | | | |  | |
| Study design | 4 | Present key elements of study design early in the paper | This retrospective study… | |  |
| Setting | 5 | Describe the setting, locations, and relevant dates, including periods of recruitment, exposure, follow-up, and data collection | Eligible participants were individuals aged 18–80 years who were diagnosed with KOA and received PRP therapy between January 2022 and November 2023 at a rehabilitation medical center. The inclusion criteria followed the national clinical guidelines (21) and required recurrent knee pain in the previous month along with at least two of the following: 1) Radiographic findings (from standing or weight-bearing views) indicating joint space narrowing, subchondral sclerosis and/or cystic changes, and osteophyte formation at the joint margins; 2) age ≥50 years; 3) morning stiffness lasting ≤30 min; 4) audible joint crepitus during movement. Additional inclusion criteria included a VAS score of ≥40/100 and receiving at least one PRP injection. The exclusion criteria included individuals diagnosed with other lower limb disorders that affected daily activities or osteoarthritis of other joints (such as the hip or ankle), PRP injection duration of less than 8 weeks (22), or those receiving other biological treatments (such as stem cell therapy or systemic immunosuppressive medications) within the past year. | |  |
| Participants | 6 | (*a*) *Cohort study*—Give the eligibility criteria, and the sources and methods of selection of participants. Describe methods of follow-up  *Case-control study*—Give the eligibility criteria, and the sources and methods of case ascertainment and control selection. Give the rationale for the choice of cases and controls  *Cross-sectional study*—Give the eligibility criteria, and the sources and methods of selection of participants | This study is a case study | |  |
|  |  | (*b*) *Cohort study*—For matched studies, give matching criteria and number of exposed and unexposed  *Case-control study*—For matched studies, give matching criteria and the number of controls per case |  | |  |
| Variables | 7 | Clearly define all outcomes, exposures, predictors, potential confounders, and effect modifiers. Give diagnostic criteria, if applicable | The primary outcomes were pain and functional assessments for 6 months after the first injection. Pain was assessed using the VAS, and function was evaluated using the Western Ontario and McMaster Universities Osteoarthritis Index (WOMAC), which is used for overall knee function assessment. The Minimal Clinically Important Difference (MCID) for both VAS (23) and WOMAC (24) is approximately 20%. The participants were classified into the effective and the ineffective groups based on the MCID of their VAS or WOMAC. Those with changes beyond the MCID were included in the effective group. The secondary outcome was safety, which was primarily evaluated through adverse event reports during the 6-month follow-up period. | |  |
| Data sources/ measurement | 8* | For each variable of interest, give sources of data and details of methods of assessment (measurement). Describe comparability of assessment methods if there is more than one group | The primary outcomes were pain and functional assessments for 6 months after the first injection. Pain was assessed using the VAS, and function was evaluated using the Western Ontario and McMaster Universities Osteoarthritis Index (WOMAC), which is used for overall knee function assessment. The Minimal Clinically Important Difference (MCID) for both VAS (23) and WOMAC (24) is approximately 20%. The participants were classified into the effective and the ineffective groups based on the MCID of their VAS or WOMAC. Those with changes beyond the MCID were included in the effective group. The secondary outcome was safety, which was primarily evaluated through adverse event reports during the 6-month follow-up period. | |  |
| Bias | 9 | Describe any efforts to address potential sources of bias | All injection procedures were performed by clinicians with over 5 years of experience in injection therapy. Standardization was ensured through uniform training in PRP preparation and application. | |  |
| Study size | 10 | Explain how the study size was arrived at | Given the observational nature of our study, we followed the recommendation from a previous study (25) to set the sample size at 5 to 10 times the number of factors. | |  |

Continued on next page

| Quantitative variables | 11 | Explain how quantitative variables were handled in the analyses. If applicable, describe which groupings were chosen and why | For normally distributed continuous variables, estimated means and standard deviations were presented. Medians and interquartile ranges (IQR) were used for non-normally distributed variables. |  |
| --- | --- | --- | --- | --- |
| Statistical methods | 12 | (*a*) Describe all statistical methods, including those used to control for confounding | Statistical analysis was performed using SPSS Statistics, version 27.0 (IBM Corp., Armonk, NY, USA) and R version 4.1.0 (R Core Team, Vienna, Austria). |  |
|  |  | (*b*) Describe any methods used to examine subgroups and interactions | Univariate analysis and multivariate analysis |  |
|  |  | (*c*) Explain how missing data were addressed | missing outcomes were not imputed. |  |
|  |  | (*d*) *Cohort study*—If applicable, explain how loss to follow-up was addressed  *Case-control study*—If applicable, explain how matching of cases and controls was addressed  *Cross-sectional study*—If applicable, describe analytical methods taking account of sampling strategy | The baseline characteristics of those that completed the trial (completers) and those who did not (non-completers) are shown in Table 1. |  |
|  |  | (*e*) Describe any sensitivity analyses | N/A |  |
| Results | | | | |
| Participants | 13* | (a) Report numbers of individuals at each stage of study—eg numbers potentially eligible, examined for eligibility, confirmed eligible, included in the study, completing follow-up, and analysed | A total of 220 participants with KOA who received PRP injections participated in this trial. Overall, 23 participants were excluded at screening and 57 were lost to follow-up after 6 months. Finally, 140 individuals were included in this study (Figure 1). |  |
|  |  | (b) Give reasons for non-participation at each stage | Figure 1 |  |
|  |  | (c) Consider use of a flow diagram | Figure 1 |  |
| Descriptive data | 14* | (a) Give characteristics of study participants (eg demographic, clinical, social) and information on exposures and potential confounders | Among the participants, 102 (72.9%) were female and 38 (27.1%) were male. The mean age of the participants was 60.4 ± 10.7 years. The median disease duration was 3 years (IQR: 1.5–6.0 years). Based on the KL grade system, 52 participants (37.1%) were graded as level I, 42 individuals (30%) as level II, 44 individuals (34.1%) as level III, and 2 individuals (1.4%) as level IV. |  |
|  |  | (b) Indicate number of participants with missing data for each variable of interest | The baseline characteristics of those that completed the trial (completers) and those who did not (non-completers) are shown in Table 1. |  |
|  |  | (c) *Cohort study*—Summarise follow-up time (eg, average and total amount) | N/A |  |
| Outcome data | 15* | *Cohort study*—Report numbers of outcome events or summary measures over time |  |  |
|  |  | *Case-control study—*Report numbers in each exposure category, or summary measures of exposure | The median (IQR) VAS score decreased from 66.5 (27) to 24 (34) at 6 months (Z = −10.159, r = −0.86, 95% CI = −38–−30.5), p < 0.001). In terms of the WOMAC score, a significant improvement was observed as the score declined from 29 (22) to 12 (14) after 6 months (Z = −9.790, r = −0.83, 95% CI = -16.5 –−12), p < 0.001). |  |
|  |  | *Cross-sectional study—*Report numbers of outcome events or summary measures |  |  |
| Main results | 16 | (*a*) Give unadjusted estimates and, if applicable, confounder-adjusted estimates and their precision (eg, 95% confidence interval). Make clear which confounders were adjusted for and why they were included | The median (IQR) VAS score decreased from 66.5 (27) to 24 (34) at 6 months (Z = −10.159, r = −0.86, 95% CI = −38–−30.5), p < 0.001). In terms of the WOMAC score, a significant improvement was observed as the score declined from 29 (22) to 12 (14) after 6 months (Z = −9.790, r = −0.83, 95% CI = -16.5 –−12), p < 0.001). |  |
|  |  | (*b*) Report category boundaries when continuous variables were categorized | N/A |  |
|  |  | (*c*) If relevant, consider translating estimates of relative risk into absolute risk for a meaningful time period | N/A |  |

Continued on next page

| Other analyses | 17 | Report other analyses done—eg analyses of subgroups and interactions, and sensitivity analyses | PRP treatment effectiveness analysis |  |
| --- | --- | --- | --- | --- |
| Discussion | | | | |
| Key results | 18 | Summarise key results with reference to study objectives | The results of our univariate analysis revealed that disease duration, sex, KL grade, and the number of injections administered significantly influenced the effectiveness of PRP treatment. However, multivariate analyses indicated that only the number of injections significantly affected pain levels, while disease duration and BMI influenced WOMAC outcomes. Specifically, PRP appeared to be more effective in pain management in individuals with shorter disease duration, lower BMI, and those who received repeated injections. This discrepancy between the univariate and multivariate analyses may be attributed to the interactions between variables and differences in model assumptions. |  |
| Limitations | 19 | Discuss limitations of the study, taking into account sources of potential bias or imprecision. Discuss both direction and magnitude of any potential bias | This study has several limitations. First, the absence of a control group limits our ability to compare the efficacy of PRP injections with other treatment methods, potentially introducing bias. Second, subjects’ compliance with the recommendation to avoid medications for up to 6 months could not be precisely controlled, which may have introduced co-intervention bias. Third, as a single-center trial, our study may be subject to center bias. Multicenter randomized controlled trials are needed to minimize selection bias and confounding factors, as well as to enhance the causal inference of the results. |  |
| Interpretation | 20 | Give a cautious overall interpretation of results considering objectives, limitations, multiplicity of analyses, results from similar studies, and other relevant evidence | Our multivariate analysis, which accounted for potential confounding factors, showed that repeated PRP injections were about four times more effective than a single injection in reducing pain. This relief in pain may be due to the sustained release of growth factors from repeated injections, which promotes cartilage repair and reduces inflammation, thereby enhancing treatment efficacy. Although repeated injections show greater improvement than a single injection, the cost-effectiveness of PRP in improving VAS… |  |
| Generalisability | 21 | Discuss the generalisability (external validity) of the study results | This relief in pain may be due to the sustained release of growth factors from repeated injections, which promotes cartilage repair and reduces inflammation, thereby enhancing treatment efficacy. Although repeated injections show greater improvement than a single injection, the cost-effectiveness of PRP in improving VAS scores after two or three injections remains unexplored. |  |
| Other information | |  | | |
| Funding | 22 | Give the source of funding and the role of the funders for the present study and, if applicable, for the original study on which the present article is based | This research did not receive any specific grant from funding agencies in the public, commercial, or not-for-profit sectors. |  |

*Give information separately for cases and controls in case-control studies and, if applicable, for exposed and unexposed groups in cohort and cross-sectional studies.

**Note:** An Explanation and Elaboration article discusses each checklist item and gives methodological background and published examples of transparent reporting. The STROBE checklist is best used in conjunction with this article (freely available on the Web sites of PLoS Medicine at http://www.plosmedicine.org/, Annals of Internal Medicine at http://www.annals.org/, and Epidemiology at http://www.epidem.com/). Information on the STROBE Initiative is available at www.strobe-statement.org.
